# CT-based micromotion analysis for tracking fracture displacement and fragment movement in trochanteric hip fractures: an agreement study

**DOI:** 10.2340/17453674.2026.45785

**Published:** 2026-06-10

**Authors:** Henrik ÅBERG, Mari HÄNNI, Olof SANDBERG, Kenneth B JONSSON

**Affiliations:** 1 Orthopaedics, Department of Surgical Sciences, Uppsala University; and Uppsala University Hospital, Uppsala; 2 Radiology, Department of Surgical Sciences, Uppsala University; and Uppsala University Hospital, Uppsala; 3 Sectra AB, Linköping, Sweden

## Abstract

**Background and purpose:**

2-dimensional radiographs may underestimate 3-dimensional fracture displacement. CT-based micromotion analysis (CTMA) enables precise quantification of interfragmentary movement in 3 dimensions. We aimed to evaluate CTMA for quantifying fracture displacement, surgical reduction, and postoperative fragment movement in trochanteric hip fractures, and assessed measurement agreement from repeated follow-up scans.

**Methods:**

We conducted a prospective cohort study including 15 patients with trochanteric fractures treated with a cephalomedullary nail at Uppsala University Hospital between 2020 and 2023. Each patient underwent CT imaging preoperatively, postoperatively, and at follow-up. Measurement agreement was assessed using 2 follow-up CT scans acquired after patient repositioning, when the fractures were healed. The femoral shaft (or nail) served as the fixed reference, and the femoral head (or spiral blade) was analyzed relative to the mirrored contralateral hip to quantify fracture displacement. Fracture movement during healing was assessed by comparing postoperative and follow-up scans.

**Results:**

12 patients had complete imaging. Measurement agreement between repeated CTMA scans showed sub-millimetric repeatability for translation and < 1° for rotation. Mean initial fracture displacement relative to the mirrored contralateral hip was 55.6° in total rotation and 48.4 mm in total translation, improving postoperatively to 17.5° and 15.9 mm, and remaining essentially stable at follow-up. Between the postoperative and follow-up scans, total movement of the femoral head averaged 5.4° (95% confidence interval [CI] 2.8–8.1) and 6.4 mm (CI 3.9–8.9). Spiral blade total translation averaged 5.4 mm (CI 3.0–7.9). Observed movement followed expected directions, with predominant femoral shortening (z-translation −4.4 mm), medialization (x-translation −3.3 mm), and slight external rotation (z-rotation +2.4°) of the femoral shaft.

**Conclusion:**

CTMA demonstrated good measurement agreement and repeatability for quantifying fracture displacement, surgical reduction, and postoperative fragment movement in trochanteric fractures.

Trochanteric hip fractures represent a significant clinical challenge and are commonly managed with internal fixation. They are among the most frequent fragility fractures in the elderly population, with incidence increasing markedly with age [1]. Despite advances in surgical techniques, complications such as non-union and malunion remain prevalent, often leading to suboptimal patient outcomes [2,3]. Outcome after trochanteric hip fracture treatment is influenced by patient factors, fracture characteristics, treatment modality, and the quality of surgical intervention. Traditional 2-dimensional (2D) radiographic methods, often employed to evaluate outcomes such as hip shortening and rotational deformity, have important limitations [4,5]. Projection of 3-dimensional (3D) structures onto a 2-dimensional plane can distort spatial relationships, while variability in patient positioning, radiographic scale, and X-ray beam angles may compromise measurement accuracy and reliability. These limitations hinder the precise assessment of fracture displacement and postoperative fragment movement.

CT-based micromotion analysis (CTMA) is a post-processing technique that analyzes sequential CT scans to measure the 3-dimensional motion of bone fragments or implants with sub-millimeter precision [6,7]. CTMA has demonstrated high accuracy in predicting long-term outcomes in arthroplasty, and has shown accuracy and precision comparable to radiostereometric analysis (RSA) across arthroplasty applications [7–9]. Additionally, CTMA has been successfully applied to measure fracture fragment motion in pelvic and radial fractures [10,11].

We aimed to evaluate CTMA as a method for quantifying fracture displacement, surgical reduction, and postoperative fragment movement in trochanteric hip fractures, and to assess measurement agreement using repeated follow-up scans of healed fractures.

## Methods

### Study design

This prospective cohort study was conducted in the orthopedic department at Uppsala University Hospital between 2020 and 2023. The study was a pilot to develop and validate a CTMA-based workflow for precisely quantifying fracture displacement, surgical reduction quality, and postoperative fragment movement, laying the groundwork for assessing mal- and non-union risk factors in hip fracture patients.

The study was reported according to GRRAS (guidelines for reporting reliability and agreement studies) and with considerations of the recent RSA/CT-RSA guideline recommendations [12,13].

### Patients

The inclusion criteria were patient age 70 years or older, an acute hip fracture planned for treatment with a cephalomedullary nail, and linguistic and cognitive ability to understand the study protocol. Exclusion criteria included previous surgery or deformity of contralateral hip, dementia or cognitive impairment, or inability to ambulate before the fracture.

All included patients were treated with a short cephalomedullary nail (Proximal Femoral Nail Antirotation, PFNA; DePuy Synthes, Oberdorf, Switzerland) with a standard nail length of 240 mm. Postoperatively, all patients were allowed weightbearing as tolerated.

The patients were given verbal and written information regarding the additional scans and outpatient visits, and the risks related to radiation exposure from CT imaging were discussed, after which written informed consent was obtained.

### CT imaging

Preoperative CT scanning was either already performed as part of the clinical work-up or performed after informed consent had been obtained and the patient had been included in the study. A postoperative CT was performed within a few days of surgery and before the patient had been significantly mobilized and discharged from the orthopedic ward. A follow-up CT was planned to be performed after fracture healing (≥ 4 months after surgery), prior to an outpatient visit. At this time, a second CT was performed after repositioning of the patient to allow for precision measurements. All CT examinations were performed in the supine position. Precision measurements were performed at follow-up, where no true fracture or implant movement was expected, to isolate measurement variability introduced by the imaging and analysis process.

The postoperative and follow-up CT examinations were optimized for micromotion analysis. Scans were performed using a dual-energy CT scanner (Somatom Definition Flash, Siemens Healthineers, Forchheim, Germany) with a detector configuration of 128 × 0.6 mm and a pitch of 0.8. A low-dose protocol (CARE Dose 4D; reference 120 kV / 50 mAs) was applied using semi-120 kV mode, with modulation of the tube current-time product (mAs) only. To minimize metal artifacts, the iterative metal artifact reduction algorithm (iMAR, Siemens Healthineers) was applied. Images were reconstructed in the axial plane with a slice thickness of 1 mm and an increment of 0.6 mm, using medium-smooth (Bf37) and medium-sharp (Br59) kernels. Iterative reconstruction was performed with ADMIRE (Advanced Modeled Iterative Reconstruction) at level 2.

Radiation exposure was assessed for the protocol-driven follow-up imaging, which consisted of 2 CT scans acquired after fracture healing, with patient repositioning between scans for precision assessment. These scans were acquired using a consistent low-dose protocol and had reliable per-examination dose information available. The cumulative effective dose for the follow-up imaging ranged from approximately 1.4 to 4.6 mSv per patient, corresponding to 0.6–2.4 mSv per individual scan. Perioperative CT examinations (preoperative and postoperative) were performed within routine clinical workflows and were therefore subject to greater variability in acquisition parameters compared with the protocol-driven follow-up examinations. When considering the study-related postoperative CT together with the protocol-driven follow-up examinations, the estimated cumulative effective radiation exposure ranged from approximately 5 to 16 mSv per patient, remaining below the maximum cumulative dose of 20 mSv approved by the Swedish Ethical Review Authority (EPM dnr 2020-00727; amendment dnr 2020-05241).

### Image analysis

**Figure 1:**
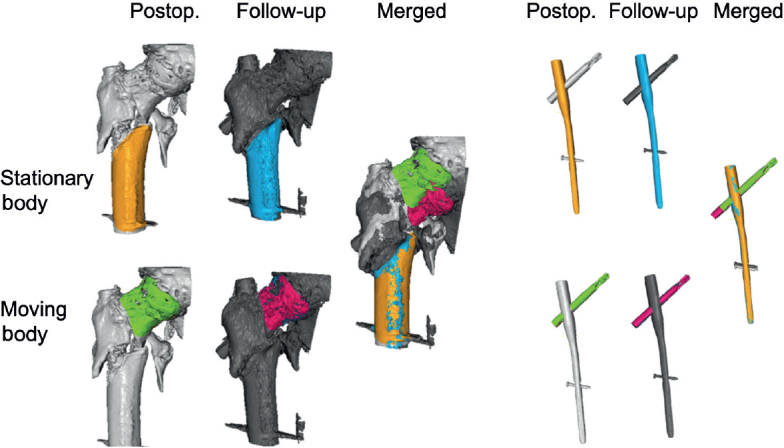
Schematic illustration of the rigid body registration used in CT-based micromotion analysis (CTMA). The stationary body (femoral shaft or nail) is fixed in space, while the moving body (femoral head or spiral blade) is analyzed relative to it. The left panel illustrates the analysis on how the femoral head moves in relation to the femoral shaft. The right panel illustrates how the spiral blade moves in relation to the nail.

The images were analyzed using the Sectra CT-based post-processing Micromotion Analysis (CTMA) software (v 26.1). Two rigid bodies were identified in each CT scan (the femoral shaft and the femoral head, or the spiral blade and the nail). The stationary body (shaft or nail) was aligned using surface-based rigid registration, while the contralateral body was measured relative to this fixed reference. Figure 1 illustrates the principle of stationary vs moving body registration as implemented in CTMA.

**Figure 2:**
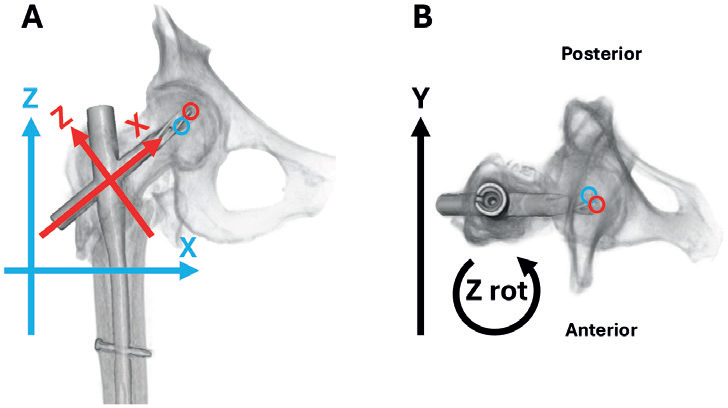
Definition of the coordinate systems and reference points used for displacement and movement analyses. (A) Frontal view. (B) Axial view from above of a right hip. The black arrow indicates the global anatomical y-axis. Blue arrows illustrate the coordinate system used to quantify movement of the femoral head relative to the fixed femoral shaft. Red arrows illustrate the coordinate system used to quantify movement of the spiral blade relative to the fixed intramedullary nail. Blue circles indicate the reference point defined within the femoral head fragment, and red circles indicate the reference point defined at the tip of the spiral blade. Positive translation was defined as movement of the reference point in the direction of the corresponding arrow. Positive rotations were defined about each axis according to the right-hand rule; positive z-rotation is illustrated by the curved arrow in panel B. In clinical terms, negative x-translation roughly corresponds to medialization of the shaft, negative z-translation to leg shortening, and positive z-rotation to external rotation of the femoral shaft.

To describe the direction of the displacement/movement, a coordinate system was first fitted on the image stack (Figure 2). This involved finding the long axis of the femoral shaft on coronal images and then rotating the perpendicular axis along the axis of the femoral neck. This fitting was performed on the injured side for measurements of initial fracture displacement and surgical reduction, and on the postoperative images for measurements of postoperative displacement. For measurements of the movement of the spiral blade the coordinate system was oriented along the long axis of the blade, such that the sliding motion of the blade in the nail was recorded as translation in the x-axis.

The displacement and movement were measured as translation along, and rotation about, the x-, y-, and z-axes of the fitted coordinate system (see Figure 2). Positive translations were defined by the direction of the corresponding axis arrows. To allow pooled interpretation across right- and left-sided fractures, the coordinate system was side-normalized such that the same sign conventions were applied to both sides. Positive rotations were defined according to the right-hand rule about each axis. Translation represents linear displacement of the moving body relative to the fixed reference body, while rotation represents angular displacement in the fitted coordinate system. Both were reported in the analysis.

The total movement for both translation and rotation were calculated using the Euclidean distance formula:


$$\text{Total}\;\text{Translation}=\sqrt{{\text{x}}^{2}+{\text{y}}^{2}+{\text{z}}^{2}}$$

$$\text{Total}\;\text{Rotation}=\sqrt{\theta_{\text{x}}^{2}+\theta_{\text{y}}^{2}+\theta_{\text{z}}^{2}}$$

where:

x, y, and z are the translational displacements/movements along the respective axes,

θ_x_, θ_y_ and θ_z_ are the rotational displacements around the axes of the fitted coordinate system.

### Measurements of initial fracture displacement and operative fracture reduction

Fracture displacement was measured relative to the anatomy of the contralateral hip. Comparative images were created by mirroring the images of the uninjured hip, which involved left–right inversion of the CT volume to match the injured side. The femoral shaft of these images was aligned, and differences in the position of the femoral heads were measured. The same procedure was applied to the postoperative and follow-up images.

### Measurements of movement of the femoral head between the postoperative time point and the follow-up time point

The images of the fractured hip from the postoperative examination were compared with the images taken at the follow-up time point. The change in the position of the femoral head from the postoperative images was determined and presented as the postoperative movement.

### Precision analysis

Precision measurements were obtained by performing 2 computed tomography (CT) scans on each patient at the follow-up stage, after the fractures had healed. Between the scans, patients were repositioned to simulate variability introduced by the CT imaging process. Additionally, each measurement involved marking the femoral head and spiral blade, and determining reference points for both, which also contributed to variability. The movements of the femoral head and the spiral blade of the osteosynthesis material were measured in relation to the femoral shaft. Translation and rotation were assessed in the x, y, and z dimensions, with total movement calculated using the Euclidean distance formula.

### Statistics

This was a descriptive methodological study aimed at developing and validating a CTMA-based workflow, and therefore no formal sample size determination was performed. The sample size was chosen to capture variability in fracture characteristics and to allow estimation of measurement agreement and repeatability. Based on previously reported precision of the method, with translation errors below 0.2 mm [7], it was anticipated that a limited number of patients would be sufficient to characterize variability at the sub-millimeter level.

For each variable, we calculated the signed paired-scan difference Δ = measurement_2_ − measurement_1_ between 2 repositioned follow-up CT scans. Measurement agreement was summarized using the mean bias (Δ), the standard deviation of paired differences (SD[Δ]), 95% repeatability coefficients defined as 1.96 × SD[Δ], and Bland–Altman limits of agreement defined as mean(Δ) ± 1.96 × SD[Δ], all expressed in original units. Lower repeatability values indicate higher measurement precision (in accordance with current recommendations for reporting agreement studies [13,14]).

Missing data was handled based on the availability of specific CT scans. 2 patients without follow-up CTs were excluded from the agreement and movement analyses, while 1 patient without a preoperative CT was excluded from the displacement analyses (see Table 1, Supplementary Figure S1). This ensured that each patient was included in the analyses for which complete data was available. 

**Table 1:** Study cohort characteristics. Classification was according to Chang et al. [5] and tip–apex distance was measured on the postoperative CT in the lateral and frontal planes

Patient	Age	Fractured side	Fracture classification	Tip–apex distance (mm)	Days to post-operative CT	Days to follow-up
1	79	Right	31a2.2	18	3	311
2	77	Right	31a3.4	14	1	274
3 **^a^**	81	Left	31a2.2	17	1	
4 **^a^**	81	Right	31a2.3	17	1	
5 **^b^**	73	Left	31a2.2	9	1	192
6	82	Left	31a3.3	12	3	142
7	83	Left	31a2.2	17	1	174
8	76	Left	31a1.3	9	1	182
9	75	Left	31a2.2	28	1	124
10	85	Left	31a2.4	11	4	129
11	83	Right	31a3.4	15	1	125
12	84	Left	31a4.1	14	3	122
13	79	Right	31a2.3	19	1	121
14	70	Right	31a4.1	17	1	124
15	83	Left	31a2.2	19	4	126

a Missing follow-up CT (excluded from precision and movement analyses).

b Missing preoperative CT (excluded from displacement analyses) due to clinical timing constraints after inclusion.

### Ethics, registration, data sharing plan, funding, and disclosures

The study was approved by the Swedish Ethical Review Authority (dnr: 2020-00727). As this was a prospective observational methodological study and not an interventional clinical trial, it was not prospectively registered in a public trial registry. The study was recorded in the local University Hospital clinical trial database.

De-identified data and analysis code are available from the corresponding author upon reasonable request and will be retained for at least 10 years at Uppsala University Hospital. AI tools were used for language editing and support in code development for data management and statistical implementation. AI tools were not used to generate results or make scientific conclusions independently. All analyses, interpretations, and final manuscript content were reviewed and approved by the authors. This study was financed by grants from the Swedish state under the ALF agreement between Uppsala University and Region Uppsala. OS is employed by Sectra AB, the vendor of the CTMA software. The study team purchased CTMA processing from Sectra; OS performed the processing and contributed to planning, figures, and editorial revisions. Sectra had no role in data collection, data interpretation, or the decision to publish. HÅ, KBJ, and MH declare no competing interests. Complete disclosure of interest forms according to ICMJE are available on the article page, doi: 10.2340/17453674.2026.45785

## Results

### Patients

We included 15 patients with a trochanteric hip fracture, of whom 13 had follow-up imaging and could be included in the agreement and movement analyses; 2 had incomplete imaging and were therefore eligible for only parts of the analysis (Table 1, Figure S1). Patient characteristics, fractured side, and fracture classification are presented in Table 1, while patient inclusion and image availability at each time point, including analysis-specific exclusions, are summarized in Supplementary Figure S1. All fractures were treated with a cephalomedullary nail, varied in complexity and displacement, and were radiologically healed at the follow-up.

### Precision analysis

Repeatability (95%) was sub-millimetric for translations and well below 1° for rotations for both the femoral head and the spiral blade across axes. Detailed agreement results included mean bias, standard deviation of paired differences, repeatability coefficients, and Bland–Altman limits of agreement (Table 2).

**Table 2:** Agreement and repeatability of CTMA measurements. These metrics quantify the agreement between repeated CTMA measurements expressed in original units

Body and movement		Mean bias (Δ)	SD(Δ)	Repeat-ability 95%	LoA lower to upper
Dimension	n				
Femoral head rotation (°)					
x	13	0.01	0.33	0.66	–0.65 to 0.66
y	13	–0.02	0.22	0.43	–0.44 to 0.41
z	13	0.07	0.38	0.75	–0.68 to 0.81
Total	13	0.48	0.25	0.49	0.00 to 0.97
Femoral head translation (mm)					
x	13	–0.05	0.19	0.37	–0.43 to 0.32
y	13	–0.04	0.22	0.43	–0.47 to 0.38
z	13	–0.02	0.11	0.23	–0.24 to 0.21
Total	13	0.26	0.17	0.34	0.00 to 0.59
Spiral blade rotation (°)					
x	13	–0.01	0.23	0.45	–0.46 to 0.44
y	13	–0.03	0.12	0.23	–0.26 to 0.21
z	13	0.02	0.22	0.43	–0.41 to 0.44
Total	13	0.31	0.10	0.20	0.11 to 0.51
Spiral blade translation (mm)					
x	13	–0.02	0.09	0.17	–0.20 to 0.15
y	13	0.03	0.23	0.45	–0.42 to 0.49
z	13	0.02	0.13	0.26	–0.24 to 0.28
Total	13	0.24	0.14	0.28	0.00 to 0.52

Mean bias (Δ) = signed paired-scan difference (Δ) (scan2 − scan1) between repositioned follow-up scans.

SD(Δ) = standard deviation of paired differences.

Repeatability 95% = 1.96 × SD(Δ).

LoA = Bland–Altman limits of agreement = mean (Δ) ± 1.96 × SD(Δ).

Total = Euclidean total across axes, which means lower LoA ≥ 0.

### Initial fracture displacement and surgical reduction

**Figure 3:**
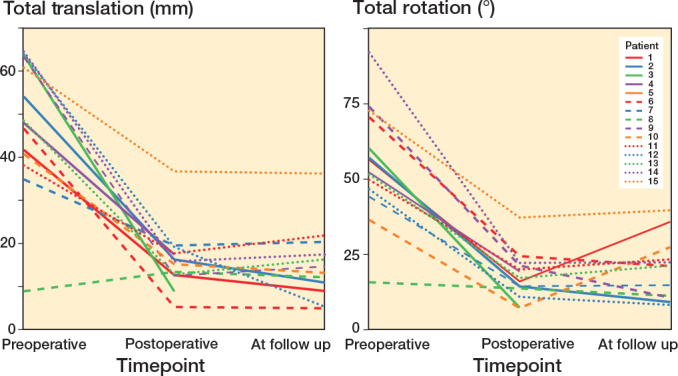
Fracture displacement over time. Each patient is represented by a separate line. Total displacement in relation to the contralateral hip was measured at 3 time points.

**Figure 4:**
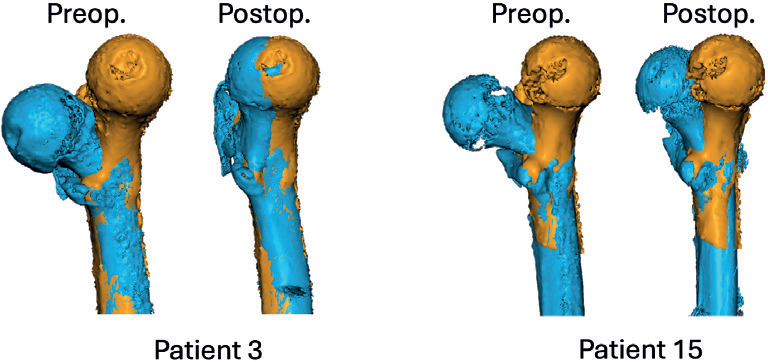
Fracture reduction quality as measured by CTMA. The fractured hip (blue) is superimposed on the mirrored contralateral hip (yellow) with the femoral shaft fixed. Patient 3: example with good reduction (low postoperative total displacement). Patient 15: example with poor reduction (higher postoperative total displacement). Each case is shown preoperatively (left) and postoperatively (right). Quantitative totals for these examples are reported in Table S1.

Initial fracture displacement was substantial, with a mean total displacement of 56° (range 15.7–91.9) in rotation and 48 mm (range 8.8–64.6) in translation. Postoperative displacement improved markedly, with mean total rotation reduced to 17.5° (range 7.1–37.1) and mean total translation reduced to 16 mm (range 5.1–36.7). At follow-up, displacement remained almost unchanged from postoperative values; rotation increased slightly by an average of 2.9° (from 17.5° to 20.3°), while translation remained stable with mean changes less than 1 mm. Displacement changes over time are illustrated in Figure 3, with examples of surgical reduction shown in Figure 4.

Displacement directions were largely consistent. For rotation, all but 1 patient showed positive rotation around the z-axis, indicative of external rotation of the femoral shaft. 1 patient (patient 6) demonstrated negative rotation around the z-axis due to a transverse intertrochanteric (31A3.3) fracture with an internally rotated distal fragment preoperatively. Similarly, translation along the z-axis was uniformly negative (shortening) except in patient 7, who had a positive z-axis translation (4.6 mm) combined with negative rotation around the y-axis, reflecting valgus alignment.

### Secondary movement during healing

**Figure 5:**
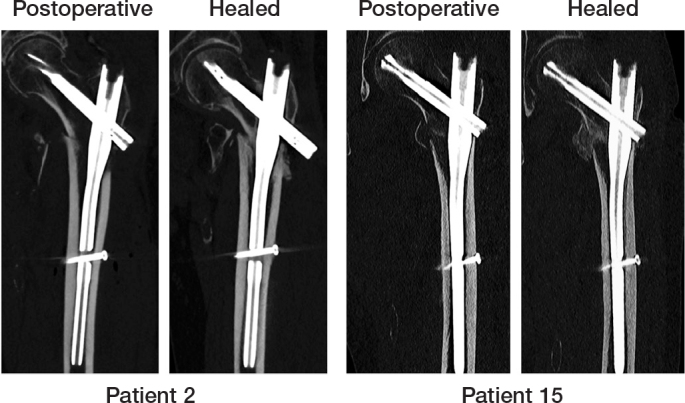
CT sections illustrating postoperative and healed images from 2 patients. The upper row shows patient 2 with the greatest observed movement between the postoperative and follow-up scans, while the lower row shows patient 15 with minimal movement. Postoperative images are shown in the left column and follow-up images after fracture healing in the right column.

In the 13 patients who underwent both postoperative and follow-up CT scans, secondary femoral head movement clearly exceeded measurement precision, as defined by the repeatability limits in Table 2. Mean total rotation was 5.4° (CI 2.8–8.1) and mean total translation was 6.4 mm (CI 3.9–8.9). Spiral blade translation was slightly less, with a mean total translation of 5.4 mm (CI 3.0–7.9) (Table 3). Examples of small and large movement between surgery and healing are shown in Figure 5.

**Table 3:** Movement of the femoral head and spiral blade between postoperative and follow-up scans. Values are shown as rotation (°) and translation (mm). Femoral head movement was measured in relation to the shaft fragment and the movement of the blade in relation to the nail

Patient	Head rotation				Head translation				Spiral blade translation	
	x	y	z	total	x	y	z	total	x	total
1	–0.89	0.41	0.52	1.11	–2.51	1.31	–0.58	2.89	–2.04	3.19
2	–1.20	3.03	6.02	6.81	–7.65	7.34	–15.11	18.46	–16.23	16.30
3	–	–	–	–	–	–	–	–	–	–
4	–	–	–	–	–	–	–	–	–	–
5	–1.63	1.71	–0.66	2.46	–5.51	0.12	–8.56	10.18	–11.98	12.01
6	–0.65	0.18	0.35	0.76	–1.39	0.81	–3.61	3.95	–1.85	2.00
7	0.80	–0.83	1.34	1.77	–2.05	1.11	–0.39	2.36	–2.56	2.61
8	–2.42	1.21	–1.70	3.21	–3.38	–0.02	–3.81	5.09	–3.88	3.89
9	12.81	1.87	11.41	17.38	–4.24	–0.79	–8.62	9.64	–7.36	7.41
10	1.99	–0.13	5.19	5.56	–0.36	5.45	–0.71	5.51	–0.38	1.24
11	–7.53	0.31	–3.79	8.44	–3.64	2.33	–3.07	5.30	–4.37	4.38
12	7.03	2.49	6.81	10.20	–6.25	–2.35	–4.84	8.25	–5.89	6.29
13	–2.26	0.82	0.67	2.49	–0.47	2.08	–2.43	3.24	–1.78	2.84
14	8.45	–1.84	3.86	9.41	–5.17	–1.13	–5.82	7.87	–7.08	7.40
15	0.53	–0.05	0.90	1.05	–0.13	0.56	–0.22	0.62	0.01	0.97
Mean	1.16	0.71	2.38	5.43	–3.29	1.29	–4.44	6.41	–5.03	5.43
Median	–0.65	0.41	0.90	3.21	–3.38	0.81	–3.61	5.30	–3.88	3.89
SD	5.36	1.35	4.11	4.90	2.41	2.63	4.29	4.63	4.73	4.49
CI lower	–1.76	–0.03	0.15	2.77	–4.60	–0.13	–6.77	3.89	–7.60	2.98
CI upper	4.07	1.44	4.61	8.10	–1.98	2.72	–2.11	8.93	–2.46	7.87

Clinical interpretation provides approximate clinical descriptors for movement directions whose 95% confidence intervals did not cross zero.

Negative z-translation of the femoral head indicates leg shortening.

Positive z-rotation of the femoral head indicates external rotation of the femoral shaft.

Negative x-translation of the femoral head corresponds to medialization of the shaft.

Negative x-translation of the spiral blade represents movement along the blade long axis.

Axis-specific analyses revealed femoral head translation primarily along the z-axis, representing leg shortening (mean –4.4 mm, CI –6.8 to –2.1), and along the x-axis, representing femoral shaft medialization (mean –3.3 mm, CI –4.6 to –2.0). Movements along these axes were uniformly consistent across all fractures. Rotational movement was greatest around the z-axis (mean 2.4°, CI 0.2–4.6), corresponding to internal rotation of the head relative to the shaft, i.e., external rotation of the shaft fragment during healing, although 3 patients had minor rotations in the opposite direction. The spiral blade exhibited marked translation only along its long axis (x-axis), with a mean translation of –5.0 mm (CI –7.6 to –2.5), consistent with sliding of the blade within the nail (see Table 3).

## Discussion

The aim of this study was to evaluate CT-based micromotion analysis (CTMA) with respect to measurement agreement for quantifying fracture displacement, surgical reduction, and postoperative fragment movement in trochanteric hip fractures treated with cephalomedullary nails. The main findings were that CTMA showed measurement agreement sufficient to support detection of clinically relevant movement. Translation repeatability thresholds were below 1 mm, whereas the mean observed post-surgical movement was 6.4 mm. In addition, the directions of both initial fracture displacement and postoperative movement were consistent with established clinical patterns, including leg shortening, external rotation of the femoral shaft, and femoral shaft medialization.

Radiostereometric analysis (RSA) has previously been used to study fracture and implant motion in trochanteric hip fractures, providing detailed descriptions of fracture behavior during healing, including femoral shortening and rotational changes [15,16]. However, the invasive nature of RSA and its limited availability have restricted its use primarily to small, specialized research cohorts. CTMA, by contrast, provides precise three-dimensional quantifications that may help identify displacement patterns of prognostic relevance. It may also offer new possibilities for studying implant placement. Tip–apex distance (TAD) is currently a widely accepted predictor of implant failure [17], but it represents only a simplified description of implant position. CTMA could enable a more comprehensive three-dimensional assessment of implant placement and help clarify whether TAD itself is the critical factor or merely a proxy for other aspects of malposition that are more strongly associated with failure. Such analyses may improve understanding of which positional factors are truly prognostic and could also support evaluation of new implant designs. Furthermore, postoperative movement measured by CTMA over time may serve as a proxy for later failure or unfavorable clinical outcomes, as excessive movement may indicate insufficient stability. CTMA might also be adapted to assess inducible movement in incompletely healed fractures, which could help identify healing disturbances by demonstrating persistent motion under different loading conditions [18].

Due to the small sample size, we were unable to analyze clinical outcomes or associations between initial fracture displacement, postoperative displacement, or TAD and subsequent fracture movement during healing. Future larger studies with sufficient statistical power should comprehensively explore these important clinical questions. Such studies could employ multivariate regression models, incorporating variables such as initial displacement magnitude, fracture type, patient BMI, and bone quality, to refine predictive models and enhance clinical decision-making.

### Limitations

The primary limitation of this study is the small sample size, which restricted our ability to analyze clinical outcomes and limits generalizability. Nevertheless, the study provides a methodological basis for future investigations in larger cohorts.

Radiation exposure remains an important issue when considering CT-based methods for evaluating fracture reduction and postoperative movement in clinical practice. In the present study, the protocol-driven follow-up CT examinations were associated with relatively low and consistent effective radiation doses on an individual level, supporting the practical use of this approach. Similar to radiostereometric analysis in arthroplasty, longitudinal assessment of fracture or implant movement over time may provide clinically meaningful information, provided that radiation exposure is kept at an acceptable level. With further protocol optimization and ongoing developments in CT technology, such as photon-counting CT, effective radiation dose may be reduced even further while maintaining sufficient image quality [19].

Additionally, some measurement variability may arise from how the coordinate system is defined across scans. In the present software implementation, the coordinate system could not be fixed to the mirrored non-injured hip as a stable anatomical reference. Instead, for displacement analyses, the coordinate system had to be defined on the fractured side for each scan. Small differences in coordinate system orientation between scans may therefore contribute to variability in the measured translations and rotations. This limitation primarily affects analyses of absolute fracture displacement relative to the contralateral hip and is less relevant for movement analyses between postoperative and follow-up scans. Addressing this limitation in future software developments could significantly enhance precision. Furthermore, metal artifacts from implants can pose challenges for image interpretation; although this was mitigated in our study through the use of titanium implants, future studies involving stainless steel implants might face greater artifact-related difficulties. Finally, a potential limitation of using the contralateral hip as an anatomical reference is that asymmetry due to degenerative changes or prior pathology on the uninjured side may complicate or preclude its use as a reliable reference. This consideration may be more relevant in future large-scale studies, where contralateral pathology cannot always be excluded.

### Conclusion

This study demonstrates that CTMA had good measurement agreement and repeatability for quantifying fracture displacement, surgical reduction, and postoperative fragment movement in trochanteric hip fractures. The method provides detailed three-dimensional information on displacement magnitude and direction that is not accessible with standard two-dimensional imaging. Further studies in larger cohorts are required to evaluate the clinical implications of these measurements and to determine how CTMA may contribute to future fracture assessment and management.

### Supplementary data

A Supplementary Table is available as supplementary data on the article page, doi: 10.2340/17453674.2026.45785

## Supplementary Material



## Notes

HÅ, KBJ, and MH planned the study and designed the methods. HÅ, KBJ, and MH handled patient inclusion and data collection. OS performed the CTMA processing and HÅ, KBJ, and OS performed the statistical analysis. HÅ, KBJ, MH, and OS drafted and critically revised the manuscript. All authors approved the final version and agree to be accountable for all aspects of the work.

The authors would like to thank Jabbar Mohammed for assistance with patient inclusion.

Handling co-editors: Bart Pijls and Robin Christensen

*Acta* thanks Rami Madanat and Stephan Maximilian Röhrl for help with peer review of this manuscript.

## References

[1] Michaëlsson K, Baron J A, Byberg L, Larsson S C, Melhus H, Gedeborg R. Declining hip fracture burden in Sweden 1998–2019 and consequences for projections through 2050. Sci Rep 2024; 14(1): 706. doi: 10.1038/s41598-024-51363-6. 38184745 PMC10771431

[2] Knauf T, Buecking B, Hack J, Barthel J, Bliemel C, Aigner R, et al. Development of the Barthel Index 5 years after hip fracture: results of a prospective study. Geriatr Gerontol Int 2019; 19(8): 809–14. doi: 10.1111/ggi.13723. 31264331

[3] Tosounidis T H, Castillo R, Kanakaris N K, Giannoudis P V. Common complications in hip fracture surgery: tips/tricks and solutions to avoid them. Injury 2015; 46:S3–S11. doi: 10.1016/j.injury.2015.08.006. 26298022

[4] Embden D van, Stollenwerck G A N L, Koster L A, Kaptein B L, Nelissen R G H H, Schipper I B. The stability of fixation of proximal femoral fractures: a radiostereometric analysis. Bone Jt J 2015; 97-B(3): 391–7. doi: 10.1302/0301-620x.97b3.35077. 25737524

[5] Chang S M, Wang Z H, Tian K W, Sun G X, Wang X, Rui Y F. A sophisticated fracture classification system of the proximal femur trochanteric region (AO/OTA-31A) based on 3D-CT images. Front Surg 2022; 9: 919225. doi: 10.3389/fsurg.2022.919225. 36117839 PMC9471135

[6] Olivecrona H, Maguire G Q, Noz M E, Zeleznik M P, Kesteris U, Weidenhielm L. A CT method for following patients with both prosthetic replacement and implanted tantalum beads: preliminary analysis with a pelvic model and in seven patients. J Orthop Surg Res 2016; 11(1): 27. doi: 10.1186/s13018-016-0360-7. 26911571 PMC4766687

[7] Brodén C, Sandberg O, Olivecrona H, Emery R, Sköldenberg O. Precision of CT-based micromotion analysis is comparable to radiostereometry for early migration measurements in cemented acetabular cups. Acta Orthop 2021; 92(4): 1–5. doi: 10.1080/17453674.2021.1906082. 33821746 PMC8381926

[8] Vusse S F V D, Laat N N D, Koster L A, Kaptein B L. The accuracy and precision of CT-RSA in arthroplasty: a systematic review and meta-analysis. Acta Orthop 2025; 96: 295–303. doi: 10.2340/17453674.2025.43334. 40159987 PMC11971844

[9] Angelomenos V, Shareghi B, Itayem R, Mohaddes M. Comparison of the CT-based micromotion analysis method versus marker-based RSA in measuring femoral head translation and evaluation of its intra- and interobserver reliability: a prospective agreement diagnostic study on 27 patients up to 1 year. Acta Orthop 2025; 96: 38–44. doi: 10.2340/17453674.2024.42705. 39786207 PMC11734532

[10] Lundin N, Olivecrona H, Bakhshayesh P, Murkes L G, Enocson A. Computed tomography micromotion analysis in the follow-up of patients with surgically treated pelvic fractures: a prospective clinical study. Eur J Orthop Surg Traumatol 2023; 33(7): 3143–51. doi: 10.1007/s00590-023-03542-w. 37059868 PMC10504208

[11] Lundqvist E, Olivecrona H, Wretenberg P, Sagerfors M. CT-based micromotion analysis after locking plate fixation of AO Type C distal radius fractures. Indian J Orthop 2023; 57(12): 2031–9. doi: 10.1007/s43465-023-01020-3. 38026840 PMC10673767

[12] Kaptein B L, Pijls B, Koster L, Kärrholm J, Hull M, Niesen A, et al. Guideline for RSA and CT-RSA implant migration measurements: an update of standardizations and recommendations. Acta Orthop 2024; 95: 256–67. doi: 10.2340/17453674.2024.40709. 38819193 PMC11141406

[13] Kottner J, Gajewski B J, Streiner D L. Guidelines for Reporting Reliability and Agreement Studies (GRRAS). Int J Nurs Stud 2011; 48(6): 659–60. doi: 10.1016/j.ijnurstu.2011.01.017. 21376316

[14] Christensen R, Ranstam J, Overgaard S, Wagner P. Guidelines for a structured manuscript: statistical methods and reporting in biomedical research journals. Acta Orthop 2023; 94: 243–9. doi: 10.2340/17453674.2023.11656. 37170796 PMC10176201

[15] Alm C E, Karlsten A, Madsen J E, Nordsletten L, Brattgjerd J E, Pripp A H, et al. No benefit of the trochanteric stabilizing plate on loss of fracture reduction in AO/OTA 31-A2 trochanteric fractures. Bone Jt Open 2024; 5(1): 37–45. doi: 10.1302/2633-1462.51.bjo-2023-0082.r1. 38240179 PMC10797560

[16] Bojan A J, Jönsson A, Granhed H, Ekholm C, Kärrholm J. Trochanteric fracture-implant motion during healing: a radiostereometry (RSA) study. Injury 2018; 49(3): 673–9. doi: 10.1016/j.injury.2018.01.005. 29397996

[17] Wittauer M, Henry J, Sánchez-Rosenberg G, Lambers A P, Jones C W, Yates P J. Evaluation of reduction quality and implant positioning in intertrochanteric fracture fixation: a review of key radiographic parameters. World J Orthop 2025; 16(8): 106982. doi: 10.5312/wjo.v16.i8.106982. 40838220 PMC12362672

[18] Wee M A T, Dobbe J G G, Kievit A J, Schafroth M U, Maas M, Blankevoort L, et al. Inducible displacement CT for implant loosening detection: a scoping review on methods, validation, and challenges. Acta Orthop 2026; 97: 136–47. doi: 10.2340/17453674.2026.45512. 41729140 PMC12927442

[19] Grunz J P, Huflage H. Photon-counting detector CT applications in musculoskeletal radiology. Investig Radiol 2025; 60(3): 198–204. doi: 10.1097/rli.0000000000001108. 39088264 PMC11801470

