# Supplementary material for: CT-based micromotion analysis for tracking fracture displacement and fragment movement in trochanteric hip fractures: an agreement study
Source: Acta Orthop. 2026 Jun 10;97:344–51. doi: 10.2340/17453674.2026.45785 (PMC13250723; doi:10.2340/17453674.2026.45785)
Supplement: Supplementary file 1 [file ActaO-97-45785-s1.pdf]

SUPPLEMENTAL TABLES

Patient	Initial Displacement								Postoperative Displacement								Follow-up Displacement							
	rotation				translation				rotation				translation				rotation				translation			
Dimension	x	y	z	t	x	y	z	t	x	y	z	t	x	y	z	t	x	y	z	t	x	y	z	t
		Varus	Ext. rot				Leg short			Varus	Ext. rot				Leg short			Varus	Ext. rot				Leg short	
1	-25.7	47.7	9.0	**56.3**	20.9	24.1	-26.9	**41.7**	-5.0	7.8	-13.3	**15.9**	11.8	-3.7	-2.1	**12.6**	-23.0	3.3	-28.0	**35.7**	8.5	-2.3	0.5	**8.9**
2	15.9	10.8	55.4	**57.0**	29.0	42.7	-16.1	**54.1**	0.4	0.2	-14.3	**14.3**	-0.7	-15.2	5.5	**16.2**	3.3	1.6	-8.3	**9.1**	-8.1	-5.8	-4.2	**10.8**
3	-7.2	35.6	46.4	**60.1**	31.4	51.3	-21.2	**63.8**	-1.7	-5.7	-4.1	**7.3**	-4.5	7.0	2.7	**8.8**	-	-	-	**-**	-	-	-	**-**
4	-21.4	26.0	35.8	**52.1**	10.6	45.8	-9.5	**48.0**	-15.6	8.7	-8.5	**19.2**	10.9	13.3	-2.3	**17.4**	-	-	-	**-**	-	-	-	**-**
5	-	-	-	**-**	-	-	-	**-**	-	-	-	**-**	-	-	-	**-**	-	-	-	**-**	-	-	-	**-**
6	-60.2	47.0	-35.1	**70.4**	18.4	17.0	-39.4	**46.7**	-10.7	-5.1	-20.8	**24.3**	1.6	-2.7	-4.1	**5.1**	-10.5	-3.8	-17.5	**21.0**	-0.3	-0.9	-4.7	**4.8**
7	-29.2	-2.4	33.9	**44.1**	0.4	34.5	4.6	**34.8**	-0.7	7.4	12.2	**14.4**	11.1	13.3	-8.8	**19.4**	-0.8	7.0	12.9	**14.7**	9.4	13.9	-11.4	**20.3**
8	-4.7	5.2	13.8	**15.7**	2.5	7.9	-2.8	**8.8**	3.7	10.3	-7.8	**13.6**	6.4	-7.1	-9.2	**13.3**	6.8	8.1	-3.2	**11.2**	1.6	-6.1	-10.2	**12.0**
9	-8.3	47.8	54.0	**73.9**	34.9	38.1	-36.6	**63.4**	-17.3	-2.7	-11.6	**21.2**	-1.4	12.2	-1.8	**12.4**	-9.7	-1.3	-3.8	**10.6**	-5.4	12.4	-5.3	**14.6**
10	2.9	24.4	27.7	**36.4**	12.4	25.9	-28.9	**40.8**	-2.7	4.4	-5.1	**7.1**	-2.6	-6.6	-13.3	**15.1**	23.6	8.4	13.0	**27.3**	0.8	6.4	-11.4	**13.1**
11	-28.0	35.7	14.1	**49.7**	16.7	24.8	-23.6	**38.1**	-2.5	-18.6	7.0	**19.9**	-13.9	9.9	4.5	**17.6**	-14.0	-18.7	1.0	**23.3**	-17.8	12.2	3.0	**21.8**
12	-28.4	30.4	15.2	**46.5**	1.5	52.3	-36.9	**64.0**	-1.1	4.1	9.9	**10.8**	12.4	10.5	-10.0	**19.1**	-2.2	5.8	5.2	**8.1**	-0.1	4.7	2.1	**5.2**
13	-3.9	30.5	40.7	**51.5**	23.0	38.8	-17.8	**48.5**	-8.3	-8.9	12.7	**17.1**	-6.1	11.1	0.8	**12.7**	-6.8	-8.8	18.6	**21.2**	-5.9	15.1	0.8	**16.2**
14	-90.0	9.6	11.5	**91.9**	18.2	61.8	-4.8	**64.6**	-15.5	-14.3	9.0	**22.1**	-6.6	12.9	6.2	**15.7**	-3.1	-17.6	13.9	**22.3**	-13.3	11.2	-0.4	**17.4**
15	-34.3	48.0	31.0	**72.6**	27.5	51.8	-16.2	**60.9**	-28.9	17.4	12.2	**37.1**	14.1	33.6	-4.6	**36.7**	-37.9	11.1	1.3	**39.5**	9.2	34.9	-3.1	**36.2**
Mean	-23.0	28.3	25.3	**55.6**	17.7	36.9	-19.7	**48.4**	-7.6	0.3	-1.6	**17.5**	2.3	6.3	-2.6	**15.9**	-6.2	-0.4	0.4	**20.3**	-1.8	8.0	-3.7	**15.1**
Median	-23.6	30.5	29.8	**54.2**	18.3	38.5	-19.5	**48.2**	-3.8	2.1	-4.6	**16.5**	0.5	10.2	-2.2	**15.4**	-4.9	2.5	1.2	**21.1**	-0.2	8.8	-3.7	**13.8**
SD	27.0	17.0	23.4	**18.5**	11.2	15.4	13.5	**15.5**	9.0	10.1	11.7	**7.7**	8.8	12.3	6.1	**7.2**	15.3	10.0	13.8	**10.2**	8.7	11.5	5.2	**8.5**
CI Lower	-37.2	19.4	13.1	**45.9**	11.8	28.9	-26.8	**40.3**	-12.3	-4.9	-7.7	**13.4**	-2.3	-0.1	-5.8	**12.1**	-14.8	-6.1	-7.4	**14.5**	-6.7	1.5	-6.6	**10.3**

Patient	Initial Displacement								Postoperative Displacement								Follow-up Displacement							
	rotation				translation				rotation				translation				rotation				translation			
Dimension	x	y	z	t	x	y	z	t	x	y	z	t	x	y	z	t	x	y	z	t	x	y	z	t
CI Upper	-8.9	37.2	37.6	**65.2**	23.5	45.0	-12.7	**56.5**	-2.8	5.6	4.5	**21.5**	6.9	12.8	0.6	**19.6**	2.5	5.3	8.2	**26.1**	3.2	14.4	-0.8	**19.9**

Table S1. Displacement over time of fractures measured preoperatively, postoperatively and at follow-up. The displacement was measured in relation to the contralateral mirrored hip. A positive rotation along the y axis is an approximation of varus displacement of the head relatively to the shaft, and along the z axis a positive value represents external rotation of the distal fragment. Similarly, a negative translation along the z axis represents leg shortening.

Body and Movement	Dimension	n	Mean Bias (Δ)	SD(Δ)	Repeatability (95%)	LoA Lower	LoA Upper
**Femoral head Rotation (°)**	x	13	0.01	0.33	0.66	-0.65	0.66
	y	13	-0.02	0.22	0.43	-0.44	0.41
	z	13	0.07	0.38	0.75	-0.68	0.81
	t	13	0.48	0.25	0.49	-0.02	0.97
**Femoral Head Translation (mm)**	x	13	-0.05	0.19	0.37	-0.43	0.32
	y	13	-0.04	0.22	0.43	-0.47	0.38
	z	13	-0.02	0.11	0.23	-0.24	0.21
	t	13	0.26	0.17	0.34	-0.08	0.59
**Spiral blade Rotation (°)**	x	13	-0.01	0.23	0.45	-0.46	0.44
	y	13	-0.03	0.12	0.23	-0.26	0.21
	z	13	0.02	0.22	0.43	-0.41	0.44
	t	13	0.31	0.10	0.20	0.11	0.51
**Spiral blade Translation (mm)**	x	13	-0.02	0.09	0.17	-0.20	0.15
	y	13	0.03	0.23	0.45	-0.42	0.49
	z	13	0.02	0.13	0.26	-0.24	0.28
	t	13	0.24	0.14	0.28	-0.05	0.52

Δ = signed paired-scan difference (scan2 − scan1).

Repeatability (95%) = 1.96 × SD(Δ). LoA = mean(Δ) ± 1.96 × SD(Δ).

Table S2. Detailed repeatability metrics for CTMA measurements.
For each variable we report sample size (n), **mean bias** (Δ), **SD(Δ)**, **95% repeatability** (1.96 × SD[Δ]), and **Bland–Altman limits of agreement** (mean[Δ] ± 1.96 × SD[Δ]). Where indicated, we also provide **mean absolute difference (|Δ|)** with its **95% CI** and the **empirical 95th percentile of |Δ|**.
Notes: Δ = scan2 − scan1 (repositioned follow-up scans). **t** = Euclidean total across axes.
